# Usage and types of mobile medical applications amongst medical students of Pakistan and its association with their academic performance

**DOI:** 10.12669/pjms.35.2.672

**Published:** 2019

**Authors:** Aliya Hisam, Muhammad Umair Shafique, Muhammad Nashit Khurshid, Ali Hamza, Muhammad Bilal Asad, Talha Shakeel

**Affiliations:** 1 *Dr. Aliya Hisam, MBBS, MPH, FCPS (Community Medicine). Associate Professor, Department of Community Medicine, Army Medical College, National University of Medical Sciences, Rawalpindi, Pakistan*; 2 *Dr. Muhammad Umair Shafique, MBBS. Final year students, Army Medical College, Rawalpindi, Pakistan*; 3 *Dr. Muhammad Nashit Khurshid, MBBS. Final year students, Army Medical College, Rawalpindi, Pakistan*; 4 *Dr. Ali Hamza, MBBS. Final year students, Army Medical College, Rawalpindi, Pakistan*; 5 *Dr. Muhammad Bilal Asad, MBBS. Final year students, Army Medical College, Rawalpindi, Pakistan*; 6 *Dr. Talha Shakeel, MBBS. Final year students, Army Medical College, Rawalpindi, Pakistan*

**Keywords:** Medical Application, Medical Applications Usage Frequency, Undergraduates, Students, Academic Performance

## Abstract

**Objective:**

To estimate the frequency of usage and types of mobile medical applications amongst medical students of Pakistan and its association with their academic performance.

**Methods:**

The is a descriptive cross-sectional study conducted in five medical colleges. It was of 6 months duration from Sept, 2017 to Feb 2018. A sample size of 448 undergraduates was calculated by using WHO sample size calculator. Sampling technique was non-probability convenient sampling. Self-constructed questionnaire was used as data collection tool. Data were entered and analysed in SPSS version 22.

**Results:**

The study included 198 male (44.2%) and 250 female (55.8%) students (448 in total). The mean age was 21.08 ±1.542 years. About 323 (72%) students whose were routinely using medical application scored 69+7% in their professional examination while 125 (28%) students, who were not using medical application scored 67±9%. The association between average usage of medical application and academic performance was statistically significant (p<0.01). Amongst the medical applications “Medical Wikipedia” had the most frequent usage i.e. 162 (36.2%) while the least frequently used app was Disease Dictionary i.e. 50 (11.2%).

**Conclusion:**

More than half of medical students who participated in the study were using medical applications on daily basis with “Medical Wikipedia” being the most commonly used applications. Association between average usage of mobile medical applications and academic performance of the students was statistically significant. A large number of students agreed that medical applications were helpful in improving their medical as well as clinical knowledge.

## INTRODUCTION

With the advent of technology, mobile phones have become a substantial part of our lives. The IBM Simon was the first phone with a touchscreen in 1992, it’s also referred as the first “smartphone” though the term was not yet coined.^1^ A mobile application is a type of application software designed to run on a mobile device like a smartphone or a tablet. Smartphone usage has spread to many settings including that of healthcare with numerous potentials and real-life benefits.^2^ The number of college students using their cell phones during class has increased over the last few years.^3^ The younger generation of technologically capable medical professionals in training, such as students and residents, harness the power of innovative applications to improve learning.^4^ The number of smartphone users is forecast to grow from 2.1 billion in 2016 to around 2.5 billion in 2019, with smartphone penetration rates increasing as well. Just over 36 percent of the world’s population is projected to use a smartphone by 2018, up from about 10 percent in 2011.^5^

Medical Applications, which can be easily downloaded onto mobiles, have an increased popularity among medical students and young clinicians.^6^ Studies report that over 85% of health professionals and medical students use a smartphone, and 30–50% use medical applications for learning and information purposes. The use of mobile technology can significantly enhance blended learning but can have a major role in supporting on-campus teaching. Smartphones have been used in educational activities to access course content, acquire information related to students’ performance, and to encourage discussion and sharing between students and teachers. It is therefore apparent that mobile devices, such as smartphones can have a significant contribution to modern health care education, since these devices might offer possibilities to enhanced teaching and learning.^7^

According to a new report, there are now more than 165,000 mobile health applications on the market, more than doubling over the past two years.^8^ Medical mobile applications provide rapid and smart access to knowledge and enable medical students to have a firm grip over their understanding and comprehension of curriculum.^9^ Health applications have been developed based on the main and necessary needs in different levels of health care delivery including diagnosis of disease, drug resources^10^, medical calculations, search of resources, clinical communications and medical education to be used by various groups of specialists, students, and patient.^11^ The field of medical applications development is very dynamic and numerous applications are being introduced to suit the needs of medical students and healthcare professionals. With the progression of time smartphones and mobile applications are replacing the traditional settings of acquiring knowledge and are offering medical students the ability to access medical information and knowledge with unprecedented ease.

There is a need to find out the frequency of smart phone medical application usage amongst medical students and how this technology is affecting their grades. Our study aimed at finding the most common types of medical applications being used by the medical students of Pakistan and its association with their academic performance.

## METHODS

This is a descriptive cross-sectional study. Following medical colleges were included for data collection i.e. Army Medical College, Rawalpindi; Islamic International Medical College, Riphah University, Rawalpindi; Federal Medical and Dental College, Islamabad; CMH Medical College, Lahore; Services Institute of Medical Sciences, Lahore. The study was conducted in 6 months from September, 2017 to February, 2018. The sample size was calculated by using WHO sample size calculator and 448 was estimated. Sampling technique was non-probability convenient sampling. Medical undergraduates were enrolled in the study.

We excluded first year medical students, those who were unwilling to participate and those who did not use smart phones. Mobile medical applications are medical devices that are mobile applications, meet the definition of a medical device and are an accessory to a regulated medical device or transform a mobile platform into a regulated medical device. A person who is enrolled in a medical college/university and studying MBBS was taken as medical undergraduate. Self-constructed questionnaire was used as data collection tool. Data were entered and analysed in SPSS version 22. Independent sample t-test was applied to assess association between average usage of mobile medical application and academic performance and a p-value of <0.05 was taken as statistically significant. Frequencies and percentages were reported and presented through tables and graphs.

## RESULTS

Mean age of the participants was 21.08±1.542 years. Out of 448 participants, 198(44.2%) were males and 250(55.8%) were females. Total of 125 (27.9%) participants were enrolled in 2^nd^ year MBBS, 124(27.7%) participants were enrolled in 3^rd^ Year MBBS, 130(29%) participants were enrolled in 4^th^ year MBBS while a total of 69(15.4%) participants were enrolled in 5^th^ year MBBS. About 323 (72%) students were routinely using mobile medical applications while 125 (28%) didn’t use these applications at all. This is illustrated in Table-I.

**Table-I:** Demographic variables of the participants.

Age (mean ± S.D)	21.08±1.542 years
***Gender n (%)***	
Male	198 (44.2)
Females	250 (55.8)
***Year of Study n (%)***	
2nd year	125(27.9)
3rd year	124(27.7)
4th year	130(29.0)
5th year	69(15.4)
***Routine medical application usage n (%)***	
Yes	323(72)
No	125(28)

**Fig.1:**
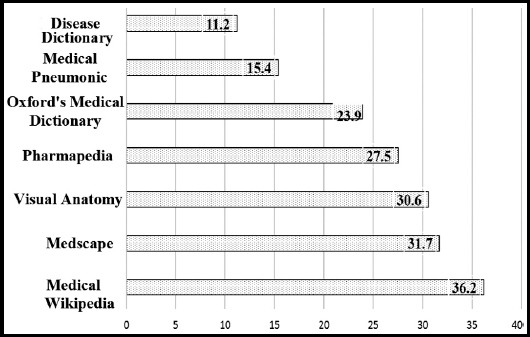
Types of medical application usage among undergraduates

Percentag of routine mobile medical application usage was found to be maximum amongst 5^th^ year medical students i.e. 93% while least amongst 2^nd^ year medical students i.e. 62%. Amongst the medical applications “Medical Wikipedia” had the most frequent usage i.e. 162 (36.2%) among undergraduates, followed by Medscape 142 (31.7%), followed by Visual Anatomy 137 (30.6%), then Pharmapedia 123 (27.5%), then Oxfords Medical Dictionary 107 (23.9%), then Medical Pneumonic 69 (15.4%) while the least frequently used app was Disease Dictionary i.e. 50 (11.2%). Details are illustrated in Fig.1.

**Fig 2:**
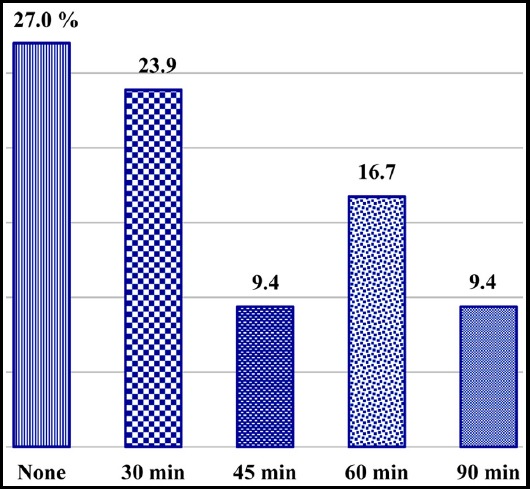
Duration of Usage of Applications per day.

Details regarding number of students and duration of mobile medical application usage per day are shown in Fig.2. Among all the enrolled participants, 266 (59.4%) students agreed that medical applications helped them improve their medical knowledge and only 77(17.2%) students disagreed with this while 105 (23.4%) students were neutral about it. On being asked whether the usage of mobile medical applications has provided easy access to understanding of the subject, 371 (82.8%) of the participants agreed to the statement, 11 (2.5%) didn’t agree while 66 (14.7%) were neutral about it. When asked abnout the usage of mobile medical applications has helped them in their medical knowledge in a particular subject, 330 (73.7%) of the participants agreed, 64 (14.23%) didn’t agree while 54 (12.1%) were neutral about it. As regards the reliability of mobile medical applications, 229 (51.1%) of the students considered these applications reliable, 33 (7.4%) didn’t agree while 186 (41.5%) were neutral about it. Responding to a question, whether the mobile medical application usage was helpful in quick revision of examination, 212 (47.3%) of the students agreed to the statement, 115 (25.7%) didn’t agree while 121 (27%) were neutral about it. When asked if they were advised by seniors to use a mobile medical application, 177 (39.5%) of the participants replied in positive while 271 (60.5%) replied in negative.

About 323 (72%) students whose were routinely using medical application scored 69+7% in their last professional examination while 125 (28%) students, who were not using medical application scored 67±9%. The association between usage of medical application and academic performance was statistically significant with a p-value of <0.01.

## DISCUSSION

The use of smart phones and their applications has increased widely over the past few years. There has been a similar change of dynamics in the field of medicine where mobile medical applications have gained popularity in current times. However, there is a variable level of interest among the medical undergraduates regarding the use of such applications for educational purposes over the world.

Frequency of usage of these smartphone applications was found to be 71% in our study which is better than a similar study conducted in Rawal Institute of Health Sciences, Islamabad^11^ in January 2015, where it was calculated to be 41.46%. In the same study, majority of students gave positive opinion about the conduction of awareness programmes in relation to medical application usage similar to our study where 57% students gave this opinion. Another similar study was performed in Karachi^12^ in July 2014, where Medical Wikipedia was found to be one of the most commonly used medical applications. This is in agreement with our study where 33.1% students had similar viewpoint. Same results were obtained in another study conducted in Multan in February 2014.^13^ A survey conducted in August 2016 on medical software applications in USA^14^ showed that 90% participants who participated in the survey thought that such applications enhance clinical knowledge which corresponds to our findings of 82% while 61% believed that they are as reliable as textbooks. However, this is less than our findings of 53%. A study in UK^15^ in October 2012, showed that 73.2% students were of the opinion that quick revision and learning is possible with medical applications. This is also in agreement with our study where majority (49%) of students had a similar point of view.

In December 2016, faculty of medicine, Jeddah^16^ conducted a similar study and found that 62.4% undergraduate’s students from 2^nd^ year to final year used mobile medical applications in course revision while 67.3% used them for looking up of medical information. These results are similar to our study findings. This specific area keeps on changing with constant change in technology and application and users need. So further studies from time to time should be conducted to assess the frequency and types of application being used and their effectiveness.

### Limitations of the study

Our study being multicentre gave better results than other similar studies. However, the use of convenient non-probability sampling technique has limitations of sample selection. The study was conducted in a short span of time.

## CONCLUSION

More than half of medical students were using medical applications on daily basis with ‘Medical Wikipedia’ being the most commonly used mobile medical application. Association between average usage of medical applications and academic performance of the students was statistically significant. A large number of students agreed that medical applications were helpful in improving their medical as well as clinical knowledge. Participants also found these applications reliable and helpful in quick revision of a test.

### Recommendations

Further studies should be done in this regard so that a systematic gateway is created in order to aid the upcoming medical professionals in confirmation of clinical diagnosis and improvement of clinical knowledge. Due to the increasing trends of mobile phone and internet, a systematic layout with respect to the awareness of usage of medical mobile applications should be carried out for better understanding of the students. Awareness programmes should be organized in order to promote the usage of such applications.
